# Proximal 10q duplication in a child with severe central hypotonia characterized by array-comparative genomic hybridization: A case report and review of the literature

**DOI:** 10.3892/etm.2014.1520

**Published:** 2014-02-06

**Authors:** EMMANOUIL MANOLAKOS, ANNALISA VETRO, ANTONIOS GARAS, LORETTA THOMAIDIS, KONSTANTINOS KEFALAS, GEORGE KITSOS, MONIKA ZIEGLER, THOMAS LIEHR, ORSETTA ZUFFARDI, IOANNIS PAPOULIDIS

**Affiliations:** 1 Laboratory of Genetics, Eurogenetica S.A., Athens 11527, Greece; 2 Department of Human and Hereditary Pathology, University of Pavia, Pavia 27100, Italy; 3 Department of Gynecology, Larissa Medical School, University of Thessaly, Larissa 41335, Greece; 4 Developmental Assessment Unit, Second Department of Paediatrics, P&A Kyriakou Children’s Hospital, University of Athens, Athens 11527, Greece; 5 Laboratory of Genetics, Bioiatriki S.A., Athens 11526, Greece; 6 Department of Ophthalmology, University of Ioannina, Ioannina 45110, Greece; 7 Institute of Human Genetics and Anthropology, Jena University Hospital, Jena D-07743, Germany

**Keywords:** 10q duplication syndrome, array-comparative genomic hybridization, developmental delay, 10q11.21→q11.22

## Abstract

Proximal 10q duplication is a well-defined but rare genetic syndrome. Duplication of proximal segments of the long arm of chromosome 10 results in a pattern of malformations, which are distinct from those of the more common distal 10q trisomy syndrome. The present study describes the case of a boy with phenotypic abnormalities (severe central hypotonia, mild ataxia, moderate developmental delay and mild dysmorphic features), due to duplication of chromosome region, 10q11.21→q11.22, which was characterized by the array-comparative genomic hybridization (CGH) technique. The phenotypic findings were compared with those in eight additional similar published cases. Major similarities have emerged, suggesting a likely minimal critical region. However, only detailed characterization of additional cases may provide firm conclusions.

## Introduction

Proximal 10q duplication is a well-defined, but rare syndrome and is often derived from a balanced translocation in a parent (1,2). In this study, the case of a boy with phenotypic abnormalities and duplication of the chromosomal region, 10q11.21→q11.22, characterized by the array-comparative genomic hybridization (CGH) technique, is reported. The phenotypic findings were compared with those in eight additional published cases with proximal partial duplication of the long arm of chromosome 10q (1–8). The partial proximal trisomy 10q consists of mild to moderate developmental delay, postnatal growth retardation, microcephaly, prominent forehead, small and deep set eyes, epicanthus, upturned nose, bow-shaped mouth, micrognathia, thick and flat helices of the ears and long, slender limbs (8). The present study concerns an 8-year-old boy with severe central hypotonia in whom array-CGH identified a *de novo* cryptic duplication of the proximal 10q.

## Materials and methods

### Cytogenetics

Conventional karyotyping based on GTG-banding (600–800 bands) was performed using standard methods on metaphases from blood leukocytes.

### Array-CGH

Molecular karyotyping was performed on DNA extracted from the whole blood of the patient and both parents. All the experiments were performed through oligo-array platforms (Human Genome CGH Microarray 44B Agilent kit; Agilent Technologies, Santa Clara, CA, USA). Briefly, 500 ng of proband and of a gender-matched pooled reference DNA (Promega Corporation, Fitchburg, WI, USA) were processed according to the manufacturer’s instructions. Fluorescence was detected in a dual-laser scanner (DNA Microarray Scanner with Sure Scan High-Resolution Technology; Model G2565CA; Agilent Technologies) and the images were extracted and analyzed through Agilent Feature Extraction software (v10.5.1.1) and DNA Analytics software (v4.0.73) (Agilent Technologies). Changes in test DNA copy number at a specific locus were observed as the deviation of the log_2_ratio value from the value of 0 of at least three consecutive probes. The quality of each experiment was assessed using a parameter provided by Agilent software (QC metric) and on the basis of DNA quality. Copy number changes identified in the samples were compared with the Database of Genomic Variants (http://projects.tcag.ca/variation/) and also visualized using the UCSC Genome Browser website (http://genome.ucsc.edu/). Moreover, DECIPHER (http://decipher.sanger.ac.uk/) and ECARUCA (http://umcecaruca01.extern.umcn.nl:8080/ecaruca/ecaruca.jsp) databases were used for comparison with possible analogous reported cases. The positions of oligomers referred to the Human Genome March 2006 assembly (hg18).

### Fluorescence in situ hybridization (FISH) analysis

FISH experiments were performed on metaphase spreads using the following as probes: Bacterial artificial chromosomes (BACs) for the chromosomal region 10q11.22 (RP11-292F22 located in 47.075–47.114 Mb, as obtained from the Children’s Hospital Oakland Research Institute, Oakland, CA, USA) and chromosome 10 centromeric probe (cep 10; Kreatech Diagnostics, Amsterdam, Netherlands). Ten metaphase spreads were analyzed for each FISH experiment. Analysis was performed using a Zeiss epifluorescent Axioskop 2 Plus microscope (Carl Zeiss Microscopy, LLC, Thornwood, NY, USA), and images were captured, enhanced and analyzed using Cytovision (Leica Biosystems, Wetzlar, Germany) software.

## Case report

The patient was a 3-year-old boy, the only child of healthy non-consanguineous parents of Greek origin (a 38-year old father and 33-year old mother at the time of birth) with unremarkable family history. The prenatal serial ultrasound examinations were reported as normal and the pregnancy was uneventful. The patient was born at 40 weeks of gestation by vaginal delivery. The Apgar scores were 8 at 1 min and 9 at 5 min. The birth weight was 2,950 g (10th centile), the birth length was 50 cm (50th centile) and the occipitofrontal circumference was 3 cm (25th–50th centile).

At 8 months the patient responded normally to age appropriate communication stimuli and parental overtures; however, he exhibited severe central hypotonia, mild ataxia and mild dysmorphic features, such as a triangular face, an enlarged cranium cerebrale, a bifid scrotum, cryptorchidism, ulnar deviation of both elbows, singe palmar creases of hands and syndactyly of the second and third toes bilaterally. Brain magnetic resonance imaging revealed hypoplasia of the corpus callosum and benign dilatation of the subarachnoid areas, while G-banding karyotyping along with detailed haematological, biochemical and metabolic examinations showed no pathology. Physiotherapy was initiated and the patient walked unaided at the age of 18 months. At 19 months, the cryptorchidism was surgically corrected. The patient first uttered words and two-word phrases at the ages of 23 and 27 months, respectively.

At 2 years and 7 months, a referral for developmental assessment was made due to language delay. The patient was a sociable boy with mild dysmorphic features that could easily have passed unnoticed, such as a triangular face, frontal bossing, micrognathia, slight auricle abnormalities, a high-arched palate, pectus carinatum, widely spaced nipples, a mild systolic murmur and a clubfoot. The patient was 15 kg (75 centile) in weight and 98 cm (90th centile) in height, and had a head circumference of 52 cm (90th centile). The developmental abilities of the patient were equivalent to the level expected at 22 months, while the patient’s speech consisted of 40 words and a few two-word phrases. Visual, hearing and heart ultrasound examinations were normal and speech therapy was initiated.

A re-evaluation was conducted at the age of 3 years. Clinical examination revealed a sociable boy with mild facial abnormalities (frontal bossing, moderate micrognathia, a gothic palate and auricular abnormalities), pectus carinatum, widely spaced nipples, a mild systolic murmur, single palmar crease and a clubfoot. The developmental abilities of the patient were further improved, being equivalent to the level at 2 years and 5 months. According to Griffiths Scales of Mental Abilities his developmental quotient was 82 (9). The patient was 17 kg (75–90th centile) in weight, 98 cm (75th–90th centile) in height and had a head circumference of 53 cm (90th–97th centile). Neurological examination revealed truncal hypotonia, but without focal signs. Informed consent was obtained from the patient’s family and the study was approved by The Second Department of Paediatrics, University of Athens, P&A Kyriakou Children’s Hospital, Athens, Greece

## Results

**Figure 1:**
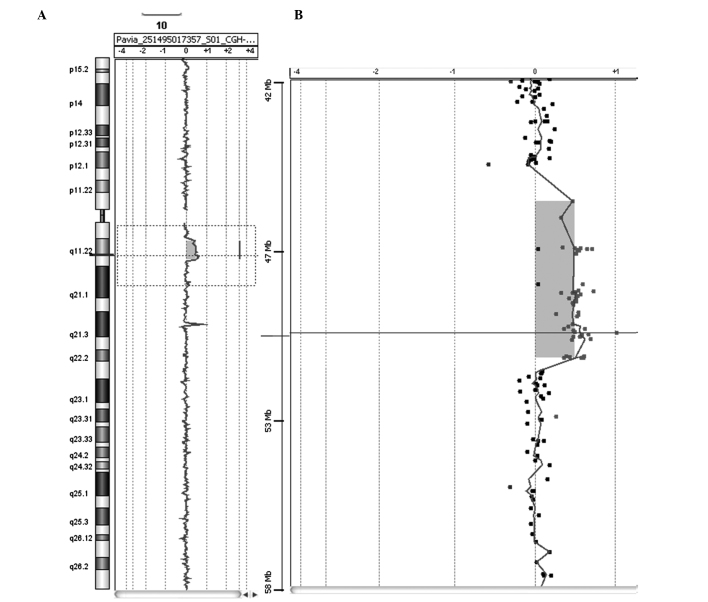
Array-CGH profile of chromosome 10 showing an interstitial duplication. (A) View of chromosome 10 and (B) the enlarged view of the rearrangement as generated by Agilent Technologies, CGH Analytics 4.0.73. The proximal duplication breakpoint was between 45.478 and 46.568 Mbp, and the distal duplication breakpoint was between 51.264 and 51.676 Mbp. The overall size of the duplication was ~5.6 Mb.

**Figure 2:**
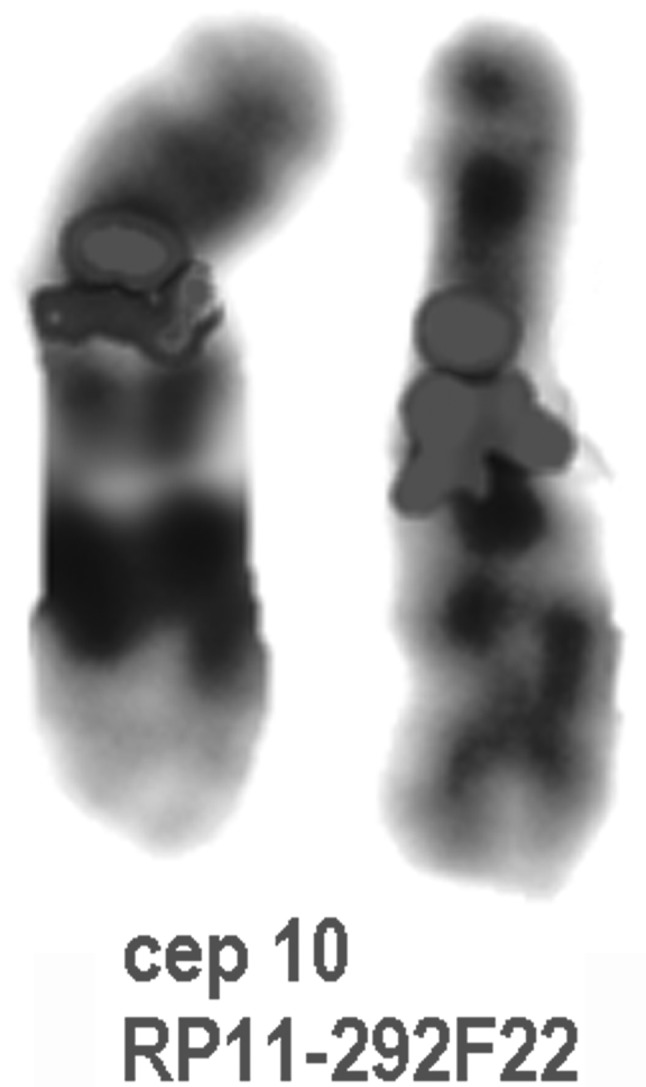
FISH analysis to confirm duplication. The analysis was performed using the probes RP11-292F22 in 10q11.22 and cep10 (centromeric probe). The resultant pattern indicated that chromosome 10 has an interstitial duplication on the proximal 10q arm. FISH, fluorescence *in situ* hybridization.

The karyotype analysis of the proband identified a normal karyotype, 46XY, and chromosome analyses of both parents were also normal. Array-CGH analysis revealed a 5.6-Mb duplication in the long arm of chromosome 10 located in the 10q11.21→q11.22 region (Fig. 1). The proximal breakpoint was observed between 45.478 and 46.568 Mbp and the distal breakpoint was between 51.264 and 51.676 Mbp. No abnormal copy number variations were identified in either parent using array-CGH technology. The DECIPHER and ECARUCA databases were used as resources to aid the genotype-phenotype correlation analysis. The duplication was confirmed by FISH analysis as shown in Fig. 2.

## Discussion

Proximal 10q duplication is a well-defined but rare genetic syndrome, often derived from a balanced translocation in a parent. The partial proximal trisomy 10q consists of mild to moderate developmental delay, postnatal growth retardation, microcephaly, a prominent forehead, small and deep set eyes, epicanthus, an upturned nose, a bow-shaped mouth, micrognathia, thick and flat helices of the ears and long, slender limbs (1–8).

The present study concerns the case of a 3-year-old boy with phenotypic abnormalities and severe central hypotonia in whom array-CGH resulted in the identification of a *de novo* cryptic duplication of the proximal 10q (10q11.21→q11.22). This is the first case of partial proximal trisomy 10q characterized by array-CGH. To the best of our knowledge, only eight cases have previously reported duplication at 10q11→10q22 (1–8). The clinical characteristics of the eight previously published cases with duplication at 10q11→q22 and the present case are outlined in Table I. In concordance, the patient of the present study showed severe central hypotonia, ataxia, a triangular face, frontal bossing, moderate micrognathia, slight auricular abnormalities, a high-arched palate, an enlarged cranium cerebrale, pectus carinatum, widely spaced nipples, a mild systolic murmur, a bifid scrotum, cryptorchidism, ulnar deviation of both elbows, single palmar creases of the hand and foot, as well as clubfoot and syndactyly of the second and third toes bilaterally. The current case did not show growth retardation and microcephaly, which have been considered common features of the syndrome (1–5,7–8). Similarly Lam *et al* (6) reported a case with proximal trisomy 10q, but without the phenotypic features of microcephaly.

The patient of the current case presented with brain malformations, which have not been identified thus far, while Lysy *et al* (8) reported a patient who presented with biliary atresia, anal anteposition and cardiac malformations.

Notably, these cases may be clearly distinguished from the cases with duplications of 10p12.1 to 10q11.22 (10). Liehr *et al* (10) identified a novel unbalanced region of chromosomal abnormalities in the pericentromeric region of chromosome 10 (10p12.1 to 10q11.22), which did not have negative phenotypic consequences.

The duplicated region identified in the present case spans only ~5.6 Mb of genomic DNA and contains ~36 genes with variable functions. Specifically these genes encode regulators of membrane organization and membrane trafficking [annexin A8 (ANXA8), ANXA8L1 and ANXA8L2], morphogenetic proteins and regulators of cell growth and differentiation [growth differentiation factor 2 (GDF2) and GDF10), transcription factors and modulators [dorsal root ganglia homeobox (DRGX)], signal transducers [mitogen-activated protein kinase 8 (MAPK8)], enzymes [choline acetyltransferase (CHAT) and poly(ADP-ribose) glycohydrolase (PARG)] and DNA repair proteins [excision repair cross-complementing rodent repair deficiency, complementation group 6 (ERCC6)]. It is worth taking into consideration that studies in rodents have suggested that the proteins, GDF2 and GDF10 are important in the differentiation of cholinergic central nervous system neurons and in skeletal morphogenesis, respectively (11,12). It is possible that the emerged severe phenotype is due to the fact that the region includes major genetic information affecting physical development, or that the duplication significantly alters the expression pattern of the corresponding genes.

Further studies are required to elucidate the pathogenic mechanisms as more cases are likely to be identified with 10q proximal duplication, which may be characterized by the advanced methodology of array-CGH. The critical region of the 10q duplication syndrome will be identified more specifically by including benign cases with duplication of 10p12.1 to 10q11.22.

## Figures and Tables

**Table I:** Summary of the clinical features in previously published cases with partial proximal trisomy 10q syndrome and the present case.

Variable	Vogel *et al* (1)	Fryns *et al* (2)	De Michelana and Campos (3)	Aalfs *et al* (4)	van Buggenhout *et al* (5)	Lam *et al* (6)	Nucaro *et al* (7)	Lysy *et al* (8)	Present case
Trisomic segment	10q11→22	10q11.2→22	10q11→22	10q11.2→22.3	10q11→22.3	10q11→22	10q11.2→22.3	10q11.2→22.3	10q11.21→11.22
Methods of confirmation	Karyotype	Karyotype	Karyotype	Karyotype FISH	Karyotype	Karyotype	Karyotype FISH	Karyotype	Karyotype FISH Array-CGH
General									
Birth weight (g)	2,200	2,650	2,400	3,750		3,000	2,830	2,720	2,950
Growth retardation	+	+	+	+	−	−	+	+	+
Developmental delay	+	+	+	+		+		+	+
Respiratory distress	+	u	u	+					
Hypertonia	u	u	+	+			+		
Craniofacial									
Microcephaly	+	+	+	+	+	−	+	+	−
Prominent forehead	−	+	−	+	+	+	−	−	+
Deep set, small eyes	+	+	+	+	+	+	+	−	+
Epicanthus	−	−	−	+	−	+	−	−	+
Strabismus	−	+	+	+	+	+	−	−	−
Iris coloboma	u	+	−	−	−	+	−	−	−
Blepharophimosis	−	−	−	−	−	u	−	+	−
Retinal dysplasia	u	+	u	−	−	+	−	−	−
Upturned nose	−	+	+	+	+	+	+	−	−
Bow-shaped mouth	+	+	+	+	+	+	+	−	+
Micrognathia	+	+	+	+	+	+	+	−	−
Highly arched palate	+	+	+	−	+	+	+	+	−
Flat, thick ear helix	+	+	+	+	+	+	+	−	+
Skeletal									
Slender limbs	+	+	−	−	−	+	+	−	+
Finger syndactyly	u	+	u	−	+	+	+	+	+
Hypermobile joints	u	+	u	−					
Rib abnormalities	+	−	u	u					

Clinical features were present (+), absent (−) or unreported (u). FISH, fluorescence *in situ* hybridization.

## References

[1] Vogel W, Back E, Imm W (1978). Serial duplication of 10 (q11 leads to q22) in a patient with minor congenital malformations. Clin Genet.

[2] Fryns JP, Kleczkowska A, Igodt-Ameye L, Van den Berghe H (1987). Proximal duplication of the long arm of chromosome 10 (10q11.2 → 10q22): a distinct clinical entity. Clin Genet.

[3] De Michelana MI, Campos PJ (1991). A new case of proximal 10q partial trisomy. J Med Genet.

[4] Aalfs CM, Hoovers JM, Nieste-Otter MA, Mannens MM, Hennekam RC, Leschot NJ (1995). Further delineation of the partial proximal trisomy 10q syndrome. J Med Genet.

[5] van Buggenhout G, Decock P, Fryns JP (1996). A distinct phenotype associated with partial trisomy 10q due to proximal direct duplication 10q11→q223?. Genet Couns.

[6] Lam FW, Chan WK, Lam ST, Chu WP, Kwong NS (2000). Proximal 10q trisomy: a new case with anal atresia. J Med Genet.

[7] Nucaro A, Faedda A, Cao A, Boccone L (2002). Partial proximal trisomy 10q syndrome: a new case. Genet Couns.

[8] Lysy PA, Sibille C, Gillerot Y, Smets F, Sokal EM (2007). Partial proximal 10q trisomy: a new case associated with biliary atresia. Hereditas.

[9] Griffiths R (1984). The Abilities of Young Children. Bucks: Association for Research in Infant and Child Development. A comprehensive system of measurement for the first eight years of life.

[10] Liehr T, Stumm M, Wegner RD, Bhatt S, Hickmann P, Patsalis PC, Meins M, Morlot S, Klaschka V, Ewers E, Hinreiner S, Mrasek K, Kosyakova N, Cai WW, Cheung SW, Weise A (2009). 10p11.2 to 10q11.2 is a yet unreported region leading to unbalanced chromosomal abnormalities without phenotypic consequences. Cytogenet Genome Res.

[11] López-Coviella I, Berse B, Krauss R, Thies RS, Blusztajn JK (2000). Induction and maintenance of the neuronal cholinergic phenotype in the central nervous system by BMP-9. Science.

[12] Cunningham NS, Jenkins NA, Gilbert DJ, Copeland NG, Reddi AH, Lee SJ (1995). Growth/differentiation factor-10: a new member of the transforming growth factor-beta superfamily related to bone morphogenetic protein-3. Growth Factors.

